# Applications of Ionic Liquids in Carboxylic Acids Separation

**DOI:** 10.3390/membranes12080771

**Published:** 2022-08-09

**Authors:** Alexandra Cristina Blaga, Alexandra Tucaliuc, Lenuta Kloetzer

**Affiliations:** “Cristofor Simionescu” Faculty of Chemical Engineering and Environmental Protection, “Gheorghe Asachi” Technical University of Iasi, D. Mangeron 73, 700050 Iasi, Romania

**Keywords:** ionic liquids, pertraction, biosynthetic products, downstream separation

## Abstract

Ionic liquids (ILs) are considered a green viable organic solvent substitute for use in the extraction and purification of biosynthetic products (derived from biomass—solid/liquid extraction, or obtained through fermentation—liquid/liquid extraction). In this review, we analyzed the ionic liquids (greener alternative for volatile organic media in chemical separation processes) as solvents for extraction (physical and reactive) and pertraction (extraction and transport through liquid membranes) in the downstream part of organic acids production, focusing on current advances and future trends of ILs in the fields of promoting environmentally friendly products separation.

## 1. Introduction

**Figure 1:**
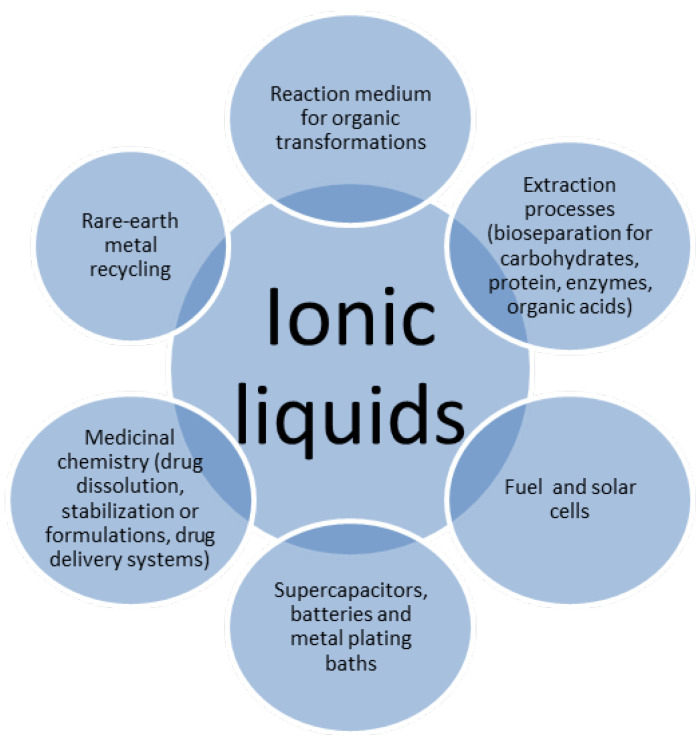
Utilizations of ionic liquids.

Ionic liquids (ILs) are tunable (polarity, hydrophobicity, H-bonding abilities) organic salts in a liquid state at room temperature (usually below 100 °C), consisting of an organic cation (variable length alkyl chain) and either an organic or a polyatomic inorganic anion [1] with many applications (Figure 1). These organic salts with outstanding properties in different fields (Figure 1) have become of major interest to scientists involved in a diverse suite of specializations [2,3,4]. Bio-based ILs have as precursors choline, glycine-betaine (N,N,N-tri (methyl) (2-dodecyloxy-2-oxomethyl)-1-ammonium docusate; N,N,N-tri (methyl) (2-dodecyloxy-2-oxomethyl)-1-ammonium thiocyanate [5]), purine and pyrimidine nucleobases (1-n-alkyl-3-methylimidazolium based nucleobases ionic liquids [6]), carbohydrates (D-xylose derived imidazolium-based chiral ionic liquids [7]), amino acids ([Cho] [AA] ILs [8]), organic acids (protic ionic liquids based on strong organic acids: trifluoracetate, methanesulfonate, and triflate of triethylammonium [9]) and can be a more economical solution for industrial separations.

The interactions between cation and anion include intra and intermolecular interactions (Coulomb forces, dipoles, π-π stackings, hydrogen bonding, van der Waals and dispersion forces) that influence the ionic liquid properties [10].

Ionic liquids were classified as room-temperature ILs (RTILs—salts derived from 1-methylimidazole that melt at temperatures below 100 °C containing different ions, e.g., [BF_4_]^−^ and [CH_3_CO_2_]^−^ in water-stable ionic liquids), task-specific ILs (TSILs—designed for separation applications), poly-ionic liquids (PILs—permanent and strong polyelectrolytes), and supported IL membranes (SILMs—thin microporous support whose pores are filled with an ionic liquid) [11]. They can be obtained through a metathesis reaction with salt exchange; several industrial producers are available (Table 1). Their use at an industrial scale is improving due to regulations imposed by governmental agencies for cleaner technologies that do not affect the environment, leading to a number of applications that have quadrupled in the last 10 years [12].

In 2021, the companies Chevron Corporation and Honeywell developed the world’s first commercial-scale process unit that utilizes ionic liquids to produce alkylate, a process used also by Tüpraş refineries (Turkey) since 2022. The process, ISOALKYTM, was first developed by Chevron in collaboration with QUILL and uses a non-volatile chloroaluminate-based ionic liquid together with an organic chloride co-catalyst, with important improvement both in product quality and yield. The collaboration between Quill and Petroliam Nasional Berhad (Petronas) made possible the development of HycaPure Hg, an adsorbent based on chlorocuprate (ii) ionic liquids, impregnated on a porous solid support, able to remove elemental mercury from gas used in the petrochemical industry. Other commercial scale utilizations of ionic liquids include: Eastman Chemical Company (Texas Eastman Division), who used a phosphonium ionic liquid (Cytec) from 1996 until 2004 for the isomerization of 3,4-epoxybut-1-ene to 2,5-dihydrofuran in the production of tetrahydrofuran, but due to low market requirement for it production has been stopped [13]. For a process that generates or uses acids, in 2004, BASF introduced the biphasic acid scavenging utilizing ionic liquids (BASIL™) process, a technology based on 1-methylimidazole addition that produces 1-methylimidazolium chloride, which can improve both phases separation and reaction yield [13].

Ionic liquids can be used for multiple functions in a process, with important properties related to tunable chirality or catalytic activity (rare in organic solvents), but are more expensive compared to organic solvents (2 to 100 times) [13]. The increasing applications in different processes will generate a higher demand with a production increase, which could reduce prices and possible competitiveness relative to traditional solvents. According to the report “Ionic Liquids: Environmentally Sustainable Solvent, Energy Storage and Separation Processes”, the ionic liquid market reached 43.0 million dollars in 2021, with an estimation of 55.8 million dollars by 2026. The main use is related to solvents and catalysts (17.8 million dollars in 2021), followed by biotechnology segment (6.0 million dollars in 2021), BASF being the main supplier. However, the use of ionic liquids as a substitute for classical chemical solvents requires closer analysis related to their biodegradability and toxicity, both as a final product and at every step of their synthesis, use and recovery/recycling, as recent studies have shown that the production of some ionic liquids can actually increase pollution (by using or generating several toxic by-products).

## 2. Green Aspects in Relation with Ionic Liquids

Compared to organic solvents with so important roles in the processes of synthesis and extraction, the use of most ionic liquids (more sustainable and benign substances) offers several advantages: high thermal stability, negligible vapor pressure (operated at high temperature and low pressure), biocompatibility (especially protic ionic liquids) and excellent solvation ability (high capacity and selectivity). Due to these properties, most of them are fit to be used in green chemistry principles: processes with reduced or no generation of harmful substances to human health or the environment [14]. The physicochemical properties of ionic liquids are diverse; one study suggested that they generate a broad variety of cations and anions and, as a consequence, highlighting not only nonvolatile, non-flammable, and stable ionic liquids but also volatile, flammable, and unstable ones [15]. The main physical properties of the most commonly used ionic liquids in biosynthetic products separations are presented in Table 2. However, some concerns related to the hydrolytic instability of [PF6]-based ionic liquids are rising, especially since its production also involves the production or use of other harmful compounds (e.g., hydrofluoric acid) [14].

They have relatively high viscosities and densities, usually above that of water, determined mostly by the cationic alkyl chain, while their water miscibility and thermal stability is in correlation with the anionic part of the molecule [20,21]. However, Zhou et al. observed that density and viscosity are influenced by both anionic and cationic parts of the ionic liquid molecule, making it possible to adjust its properties by choosing a specific substituent. For example, a longer alkyl chain at the 3-position of imidazolium will generate lower density and higher viscosity [22].

One important requirement for an extraction system applicable for biosynthetic compounds (dissolved in water in low concentrations) is that the ionic liquid is hydrophobic, in order to obtain the two phases. Water-soluble hydrophilic ionic liquids are used in aqueous biphasic systems (ABS) with suitable salting-out agents as extracting solvents. In relation to anionic charge density, ionic liquids can be either hydrophilic or hydrophobic, extracting polar but also non-polar solutes. The ionic liquid hydrophobic characteristics can be influenced by the anionic or cationic alkyl chain length [23]. IL viscosity varies between 20 and 40,000 cP, being influenced by the size of the ionic chains but also by intermolecular interactions (high interaction energy as van der Waals or electrostatic interactions, determines high viscosity). The increase in anion size determines the decrease in viscosity, while the increase in alkylic chain of the cation increases viscosity (due to stronger van der Waals interactions) [24]; a viscosity that would allow intensive mixing is important for a good mass transfer.

Experimental screening in search of an ideal solvent for a particular process can be time-consuming and costly, since there are so many available ionic liquids, but due to their configurable design and physical properties determined by choosing a specific cation and anion, ionic liquids can be good solvents for many organic compounds [25].

Ionic liquids recovery, after extraction, is favorable due to low vapor pressure, non-flammability and chemical and thermal stability, all of which makes them environmentally sustainable compared to organic solvents [26]. However, several issues have been raised regarding their biodegradability and toxicity, especially related to their recovery and recycling if they are used in large quantities (as traditional solvents). Moreover, the impact of their synthesis process on the environment is not considered in terms of the substances used (raw materials) or produced (intermediate materials: volatile organic compounds), which can be toxic both for environment and human health. Also, the energy consumed in the process and generation of waste or polluted water must be considered since the synthesis process is often complex involving a large number of steps [14]. In order to improve these issues, several alternatives have been investigated: the use of microwaves or ultrasounds as alternatives for reflux heating, which allows the significant reduction of both reaction time and the use of organic solvents [13,27]. One other possibility analyzed was the use of renewable sources [28,29] for ionic liquid production: amino acids [30,31,32,33,34,35,36], polysaccharides (cellulose, chitin, and starch, but also fructose, glucose, galactose and arabinose obtained from polysaccharides) [37,38,39,40,41,42,43,44], fatty acids [45,46,47,48,49], organic acids [9,50,51,52]. The synthesis of this type of bio-ionic liquids, involves less steps but requires the use of large amounts of toxic solvents and the yield varies between 35 and 85%, so further studies are still required [14].

The recovery and reuse of ionic liquids is very important as their price is quite high, so the chosen process must be inexpensive and eco-friendly. Several techniques can be applied for this purpose: distillation under vacuum; evaporation under reduce pressure; liquid–liquid extraction using water (for recovery of ILs from hydrophilic media) or organic solvents (for solutes immiscible with water), crystallization, decantation (BASIL and Difasol industrial processes), centrifugation, combined filtration and evaporation processes, and electro dialysis with ultrafiltration [14]. The efficiency of the regenerated ionic liquid is in the range 78–100%, but from an economic point of view, the process requires a recovery yield higher than 96%, the use for at least 10 operations, and a low cost for the recovery.

A very important issue related to the use of ionic liquids is their biodegradability, which is strongly influenced by the cation and anion nature: side chain size, central ring structure and functional group’s structure, with a more pronounced effect of the cationic part of the molecule [53,54,55,56,57,58]. The increase in the alkyl chain from the cation and the presence of functional groups as hydroxyl, carboxylic, alcohol or ether, as well as the presence of natural building blocks from renewable sources improve biodegradability [58]. Several studies showed that biodegradation can be influenced by the introduction of an appropriate substituent or core cation manipulations [56,59].

## 3. Biosynthetic Products Separation Processes Using Ionic Liquids

### Solvation Properties of Ionic Liquids

The selection of an extraction step in the downstream process of biosynthetic products is based on solute physical and chemical properties, its location (intra or extracellular), the imposed degree of purity and the cost of the extracted product [60]. For the quantitative analysis of ionic liquids molecular solvation properties, the linear solvation energy relationship (LSER) equation developed by Abraham and Taft et al. can be used. The equation provides correlations between a given solubility property (e.g., partition coefficients), with several terms that consider specific solubility interactions [61]:

$${\mathrm{logK}}_{L}=\;c+r\cdot R^{2}+s\cdot \pi^{H}+a\cdot \alpha^{H}+b\cdot \beta^{H}+l\cdot \log L$$

where: 

log K_L_—the solubility coefficient

R^2^—excess of molar refraction calculated from the solute refractive index, 

π^H^—the solute dipolarity/polarizability,

α^H^—the solute hydrogen bond acidity (measures solvent’s ability to act as a hydrogen bond donor) 

β^H^—the solute hydrogen bond basicity (measures solvent’s ability to serve as a hydrogen bond acceptor)

α, β, π are temperature-dependent solvent polarity scales (Kamlet-Taft parameters)

L—the solute–gas partition coefficient at 258 °C. 

c—a constant, 

s—denotes dipolarity/polarizability, 

a—denotes hydrogen bond basicity, 

b—denotes hydrogen bond acidity,

l—describes dispersion forces.

Lee modified this equation in terms of free energy transfer by introducing the solute internal energy term into the LSER equation. The values obtained using this equation proved that all parameters: dispersion, polarity, acidity, basicity, and molar volume, influence in equal proportions the organic solutes solvation in ionic liquids, but also that the solubility of polar compounds with aromatic groups can be increased by higher dispersion interaction compared to aqueous solutions [62].

For polarity estimation of ionic liquids, different scales have been used; the Hildebrand solubility parameter, relative permittivity, ET (30) value (cannot be used for opaque or for ionic liquids in which betaine dye no. 30 is not soluble) and the hyperfine coupling constant (AN) have been applied to ILs to provide quantitative evaluation of the polarity of ILs [25]. ET (30) value is widely used to reflect the polarity of ILs, but also their hydrogen bond donating ability [63]. According to the ET (30), the polarity of many ILs is in the range 42–63 kcal/moll, as most ionic liquids have ET values of about 60: [BMIM] [PF6]—52.3 kcal/moll [64].

The polarity decreases in the following order:

acetate > benzoate > dimethylphosphate > Cl− > Br− > NO_3^−^_ > trifluoroacetate > N(CN)_2^−^_ > C_2_H_5_SO_4^−^_ > CH_3_SO_4^−^_ > I− > CF_3_SO_3^−^_ > SCN− > ClO_4^−^_ > C(CN)_3^−^_ > NTf_2^−^_ > BF_4^−^_ > PF_6^−^_.

For identical substituents in the cation (the attached substitutions to the charged center), the polarity decreases in the order:

ammonium > imidazolium > pyridinium > pyrrolidinium [65]

Ionic liquids have been applied for the liquid–liquid extraction of metal cations, but also for organic molecules such as alcohols: ethanol, propanol, and butanol [66,67,68,69,70], organic acids: propionic acid, butyric acid, volatile fatty acid; acetic, propionic, butyric, valeric and caproic acid, etc. [71,72,73,74,75]; and proteins [76,77,78,79], aminoacids [80] and carbohydrates [1,81,82].

## 4. Carboxylic Acid Extraction Using Ionic Liquids

Carboxylic acids are used on a large scale in the chemical, pharmaceuticals, cosmetic and food industry as platform chemicals with many applications in bio-based large-volume industrial chemicals and polymer production, many of them being produced from carbohydrates or other renewable raw materials by fermentation. On a large scale, biosynthesis is already applied for the production of citric, lactic, itaconic, gluconic, 2-keto-L-gulonic and succinic acids. For other acids (adipic, malic, acrylic, 3-hydroxipropinic, pyruvic) research is in advanced stages and will probably be available in the next years [83,84,85]. The acid separation from the fermentation broth is however the most energy and cost intensive (30–40% of the total production costs) production step, requiring the development of robust, efficient separation processes [86]. Moreover, for an industrial process, the recovery part has to assure that the product purity offers a minimum of 90% yield, and low chemicals and energy consumption and low waste generation [87]. Extraction using ionic liquids (due to their superior properties over classical solvents) can be an important alternative to electrodialysis, ion exchange, membrane separation, distillation, liquid–liquid extraction, and reactive extraction for carboxylic acids recovery from fermentation broth.

Carboxylic acids are found in aqueous fermentation broth in two forms according to their dissociation constant: at pH < pKa (acid dissociation constant), the acid is undissociated, RCOOH, while at pH > pKa, the acid dissociates according to equation:

$${\mathrm{RCOOH}}_{\mathrm{aq}}\;↔\;{{\mathrm{RCOO}}^{-}}_{\mathrm{aq}}+H^{+}$$

When the aqueous solution containing the organic acid is mixed with the organic phase (pure ionic liquid or solvent containing ionic liquids), the acid can be physically extracted by solubilization and diffusion (the overbar shows molecules in the organic phase)

$${\mathrm{RCOOH}}_{\mathrm{aq}}\;↔\;\bar{\mathrm{RCOOH}}$$

or it can form complexes involving one or more molecules (n,m) of acid or ionic liquids:

nRCOOH_aq_ + m (IL) ‾↔ RCOO_n_IL_m_

These associations can be obtained as reverse micelles (the ionic liquid act as surfactant due to asymmetrical structure with localized polar charged domains and prolonged cationic alkyl chain [88,89]), which influences water solubility by including it and are broken once the extracted acid concentration increases, or as IL-acid complexes that can exist as clusters due to electrostatic and intermolecular forces [90]. To estimate the ionic liquids phase forming ability, the Kamlet–Taft parameters (a scale based on linear solvation energy relationships measuring solvent hydrogen bond acidity, α—hydrogen bond donating ability, and basicity, β—hydrogen-bond accepting ability, while π* measures solvent dipolarity and polarizability) are usually used [91].

For the extraction quantification, the distribution coefficient is used defined as the ratio between the organic acid concentrations in the organic phase (extract—IL or solvent with IL) and the raffinate phase (exhausted initial aqueous solution)

$$D=\frac{\left[\bar{\mathrm{RCOOH}}\right]}{\left[{\mathrm{RCOOH}}_{\mathrm{aq}}\right]}$$

Martak and Schlosser (2019) presented the reactive extraction mechanism for carboxylic acid, with a focus on lactic and butyric acid, considering three sub-mechanisms [88]:

(a) Competitive extraction of carboxylic acid and water (carboxylic acid and water compete for H-bond sites in the polar domains of IL)

(b) Non-competitive mechanism of carboxylic acid extraction

(c) Co-extraction of water with carboxylic acid

According to the first (competitive extraction—that considers water saturated ionic liquids), reactive extraction of carboxylic acid, RCOOH, using IL implies the formation of complexes containing *n* molecules of acid and one ion pair of IL

(1) $$\mathrm{RCOOH}+\bar{{\left(\mathrm{RCOOH}\right)}_{n-1}\left(\mathrm{IL}\right){\left(H_{2}O\right)}_{k_{n-1}}}↔\bar{{\left(\mathrm{RCOOH}\right)}_{n}\left(\mathrm{IL}\right){\left(H_{2}O\right)}_{k_{n}}}+(k_{n-1}-k_{n})H_{2}O$$

where k_n_—stability constants (equilibrium constant), characterizing the stability of the bond between the acid and IL in complexes (1, 1) and (2, 1), and between two acids in complexes with n > 2; its values systematically decrease with the increase in extracted acid molecules (n) [88,89].

For ILs that contain phosphinate and carboxylate anions, in this case, water is associated with two H-bonding sites located on the oxygen atoms from the above-mentioned IL anions functional groups (carboxylate or phosphinate). This mechanism implies the replacement of these water molecules (accumulated in polar domains of the IL) by the carboxylic acid through a competitive mechanism in order to form a complex ionic liquid-carboxylic acid, involving n molecules of acid. For carboxylic acid reactive extraction, hydrophobic ionic liquids are usually chosen; however, even if they have minimum water solubility (e.g., phosphonium IL (CYPHOS^®^ IL 104)—water solubility 9.1 g/m^3^), these ionic liquids are hygroscopic (absorb water from air) and are able to dissolve as much as 15.3 mass% of water at 25 °C [92]. The water solubility is influenced by both cation and anion structure and is characterized by strong H-bonds in the ionic liquid polar domains and, at low ionic liquid concentration, the appearance of reverse micelles in solutions [93]. Water co-extraction is influenced by the ionic liquid concentration, suggesting water bridges formation between acid chains connecting two IL ion pairs. These particularities determine that both water and carboxylic acids are being extracted through H-bonds formation by ILs. However, for IL anions that are water soluble (chloride), an ion-exchange mechanism (chloride is changed for the carboxylic acid anion) is possible, while for a hydrophobic anion, the extraction will be based on hydrogen bonds established between IL and the un-dissociated form of the carboxylic acid (aqueous phase pH lower than pka—acid dissociation constant) [91].

For the second mechanism considered, if k_n_ = 0, the complex formed does not involve water

(2) $$R-\mathrm{COOH}+\bar{{\left(R-\mathrm{COOH}\right)}_{n-1}\left(\mathrm{IL}\right)}↔\bar{{\left(\mathrm{RCOOH}\right)}_{n}\left(\mathrm{IL}\right)}$$

When the carboxylic acid is a stronger acid than the hydrophobic acid (e.g., lactic acid extracted with phosphonium IL) from the anionic part of the ionic liquid, this can be exchanged with extracted acid and the hydrophobic acid from the anion is kept in organic phase [89]. The reactive extraction mechanism of carboxylic acids is influenced by aqueous solubility of the IL anion, which can determine the formation of carboxylic acid-ionic liquid complexes that include multiple acid molecules—overloading—strongly influenced by the acid concentration (which also influences the ionic liquid water content: an increased acid concentration determines the decrease) [92].

For the third mechanism, the total equilibrium loading of IL by water in the organic phase considers not only the water directly associated with the IL (that competes with the carboxylic acid) but also the water associated with the hydrated acid (co-extracted, linearly dependent on the amount of extracted acid). For lactic acid reactive extraction using Cyphos IL-104 as ionic liquid, the number of water molecules involved in the IL-RCOOH complex formation varied from 8, in the case of low lactic acid loading, in reversed micelles, to 2, corresponding to hydrated complexes (obtained at high LA loading) [94,95]. Several carboxylic acids have been separated using ionic liquids (Table 3).

For butyric acid, reactive extraction using [P6,6,6,14] [Phos] trihexyl tetradecyl phosphonium di-2,4,4 trimethylpentyl phosphinate and [CnCnCnC1N] [Phos], trialkylmethylammonium bis (2,4,4-trimethylpentyl) phosphinate resulted in a distribution coefficient equal to 80 for low concentrations of butyric acid, the formed complex involving 12 water molecules per ion pair of the IL [109].

For nicotinic acid extraction from aqueous phase using [C_6_mim]ClO_4_, a higher extraction ability was obtained compared with the traditional solvents in the following conditions: extraction temperature of 25 °C; nicotinic acid concentration of 0.10 g/L; extraction time of 1.0 min, and equal volumes of organic and aqueous phases. The driving forces for the extraction process were found to be hydrogen bonds (main driving force), π-π interactions and hydrophobic effects. Analyzing other ionic liquids for the extraction, a decrease in the efficiency was obtained with IL cation side chain length increase, due to steric hindrance. Fan et al., 2019 analyzed the nicotinic acid re-extraction from the ionic liquids using 0.1 moll/L HCl, obtaining 91.7% recovery of nicotinic acid from the IL phase (reusable after 4 h drying at 70 °C without any extraction ability loss) [108].

For butyric acid, the distribution coefficient is higher than 80 in the extraction system using Cyphos IL-104 and lower acid concentrations, with the formation of complexes that involve one molecule of ionic liquid. When the ionic liquid was dissolved in dodecane, the extraction depends on butyric acid concentration: at high values, the process is mainly based on physical extraction, while at low butyric acid concentrations, the extraction occurs by the formation of stoichiometric acid-ionic liquid complexes [99].

For lactic acid extraction, the same ionic liquid was investigated: trihexyl (tetradecyl) phosphonium bis 2,4,4-trimethylpentylphosphinate (Cyphos^®^ IL-104), in n-dodecane, obtaining a value equal to 80, for low lactic in aqueous systems, the extraction mechanism being based mainly on hydrogen bonds, with the formation of (LAH)p(IL)(H_2_O)_2_ complexes, where *p* is between 1–3, the major complex involving 2 lactic acid molecule: (LAH)_2_(IL)(H_2_O)_2_, for 0.2 to 2 kmol/m^3^ acid concentrations. The formation of reverse micelles unstable at high temperatures was noted due to high water co-extraction in the organic phase. The authors noted a reduction in solvent viscosity from 49.4 to 33 mPa·s by increasing the temperature from 25 to 35 °C, and from 33.2 to 26.2 mPa s for the water saturated solvent [94]. Çevik et al., 2022 used 1-Butyl-3-methylimidazolium hexafluorophosphate (hydrophobic ionic liquid) in which tripropylamine (TPA) was dissolved for lactic acid separation obtaining a distribution coefficient of 255 for extractant (TPA) concentration of 1.4 moll/L, higher than in 1-octanol (212:33) and 2-octanone (141:22). The extraction efficiency, E, reached 99.61%, and loading values between 0.91 and 1.59, for initial lactic acid concentration in the aqueous phase of 1.28 moll/L, and the following reactive extraction conditions: phases mixed at 170 rpm at 298.15 K for 2 h [94]. Oliveira et al., 2012 analyzed a two-step extraction process for three short chain carboxylic acids: lactic, malic, and succinic acids, and tetradecyltrihexyl phosphonium decanoate [P66614] [Dec], the partition coefficient being 7.3 for malic acid (extraction efficiency, E = 89.4%), 10.6 for succinic acid (E = 91.4%) and 20.9 for lactic acid (E = 98.4%), after the second extraction step. However, re-extraction of the acid from the ionic liquid was not possible by distillation; a two-fold excess of sodium hydroxide aqueous solution was necessary for partial recovery of acids as sodium carboxylate [97].

Glycolic acid extraction was carried out using a mixture of 1-butyl-3-methylimidazolium hexafluorophosphate and tripropylamine (1.75 mol/L concentration), which yielded for 1.57 moll/L acid concentration a 410.60 value for the distribution coefficient and 99.76% extraction efficiency and no overloading [102].

The use of hydroxide-based ionic liquids (Tetramethylammonium hydroxide—25% solution in water, [N1111][OH], Tetrabutylammonium hydroxide—40% solution in water, methyltributylammonium hydroxide—20% solution in water, choline hydroxide—20% solution in water, [Ch][OH], and tetrabutylphosphonium hydroxide—40% solution in water) dissolved in dodecane was investigated for naphthenic acids (a mixture of cyclic, aromatic, and linear monocarboxylic acids) recovery from highly acidic model oil. The obtained results proved that the deacidification process is reduced with the increase in alkyl groups in the tetraalkylammonium ILs, due to the polarity decrease, obtaining the following order for extraction efficiency: [N1111] [OH] > [Ch] [OH] > [N1444] [OH] > [N4444] [OH] > [P4444] [OH]. The complete separation of naphthenic acid (100% acid removal from oil) was obtained at a ratio of 0.0075 IL/oil (*w*/*w*), a stirring rate of 500 rpm and 1 h reaction time [110]. Geng et al., 2022 analyzed the use of N-alkyl imidazolium carbonate ionic liquids: 1-ethyl-3-methylimidazolium acetate ([Emim]Ac), 1-ethyl-3-methylimidazolium nitrate ([Emim]NO_3_), 1-ethyl-3-methylimidazolium hydrogen sulfate ([Emim]HSO_4_), for naphthenic acid separation. The best results for the separation were obtained for the use of [Emim]_2_CO_3_ and: 40 °C temperature, 0.010 IL/oil mass ratio, 500 rot/min stirring speed and 1 h contact time. The ionic liquid aqueous solutions obtained after naphthenic acid re-extraction with hydrochloric acid and regeneration using an anion-exchange resin could be effectively reused, proving process feasibility [111].

## 5. Carboxylic Acid Separation by Pertraction Using Ionic Liquids

Pertraction or extraction and transport through liquid membranes implies the use of an organic solvent that acts as a semi-permeable liquid layer that allows the selective transport of a solute (carboxylic acid) between the feed phase and the stripping phase [112]. This method combines in one equipment two processes: extraction and stripping, with many advantages including low capital and operating cost, technical simplicity, high selectivity and the possibility to transport a solute from a dilute solution (initial phase) into a concentrated one (stripping solution) by maintaining an adequate difference between these solutions regarding the driving force (pH gradient or ionic strength) without needing a large quantity of organic phase [113].

Liquid membranes are homogeneous membranes that operate on the principle of dissolving a solute at the contact interface between the initial aqueous phase and the liquid membrane and releasing it at the interface between the membrane and the final aqueous phase, based on the concentration gradient between interfaces. While solvent extraction is an equilibrium process, extraction is governed by the kinetics of membrane transport. The amount of solute transported is not proportional to that of the membrane phase, as in the case of extraction the liquid membrane is only a short-term mediator. However, in order to select the membrane solvent, its properties must be considered: hydrophobicity to ensure immiscibility with aqueous phases, viscosity to ensure high rates of mass transfer (solute diffusion) and density, vapor pressure, but especially the dielectric constant, because one of the most important parameters that control the separation performance is the polarity of the solvent.

There are three main types of liquid membrane [114]: emulsion—ELM, supported—SLM and bulk—BLM. The first type of liquid membrane obtained by emulsification, ELM, implies intense phase mixing (5000–10,000 rpm) between the solvent and the aqueous phase in which the re-extraction takes place (internal phase), followed by dispersion of this emulsion (stabilized by a film of surfactant adsorbed at the interface between fluids) in gentle stirring conditions (200–400 rpm) in the aqueous phase, from which the solute is extracted (external phase). A supported liquid membrane (SLM) involves including an immiscible liquid solvent within the pores of an inert hydrophobic polymeric material, or inside a fibrous material. Bulk liquid membrane (BLM) implies three separated layers (generated by the solubility difference): aqueous feed and stripping phase are separated by a layer of organic solvent (the liquid membrane phase) and are based on diffusivity. The last type implies the use of special extraction equipment (with concentric cylinders, U or H-shaped, with solvent film discs, etc.).

**Figure 2:**
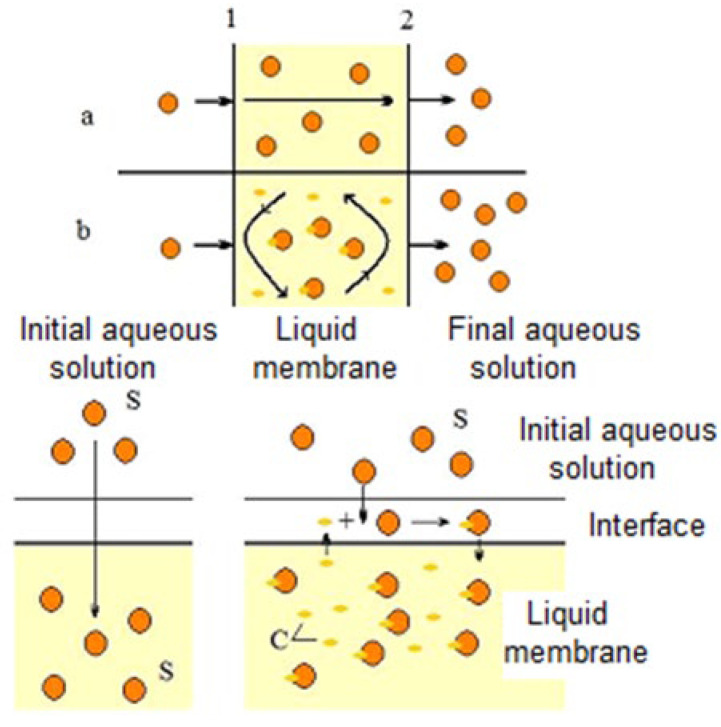
Schematic representation of liquid membrane separation processes (detailed description provided in text), S—solute, C—carrier.

The separation mechanism in liquid membranes is presented in Figure 2.

In the case of extraction by liquid membranes, the mechanism is broadly based on that of liquid–liquid extraction [85]. The general mechanism of extraction, which includes extraction, simple or facilitated transport through the membrane, and re-extraction of the solute, shown in Figure 2, involves the following steps

1. Physical (a) or reactive (b) extraction of the solute at the separation interface (1) between the initial aqueous phase and the liquid membrane. In the first case, the transfer is based only on the phenomena of solubilization and diffusion of the solute through the membrane, while in the second case (facilitated extraction), the solute is solubilized in the liquid membrane by reaction with an ionic liquid (carrier).
2. Diffusion of the solute or complex formed as a result of the interfacial reaction between the solute and the carrier from the interface (1) to the interface (2), through the liquid membrane
3. Re-extraction of the solute at the separation interface (2) between the organic phase and the final aqueous phase, with the regeneration of the solvent and the carrier.

The emulsion liquid membranes are simple and efficient ways to separate low concentration biomolecules, as carboxylic acids obtained through fermentation. Highly volatile organic solvents (hexane, dichloromethane, etc.) need to be replaced with biocompatible solvents (e.g., vegetable oils with ionic liquids as carriers) in order to have a more environmentally friendly process. Purtika and Jawa (2022) have investigated the effect of ionic liquid incorporation in a vegetable oil (sunflower, groundnut, rice bran, soya bean, olive, mustard, and coconut oil) liquid membrane. The ionic liquid, 1-butyl-3-methylimidazolium chloride ([BMlm]Cl) dissolved in rice bran oil in a concentration of 0.1% *w*/*v*, the Span 80 surfactant (1.5% (*v*/*v*)) and internal phase reagent (0.1 N NaOH concentration), 0.4 (*v*/*v*) phase ratio demonstrated to be the most stable ELM (maximum stability 165 min and complete phases separation after 1200 min). The highest ELM static stability obtained by using rice brain oil is correlated to its 59.3 cP absolute viscosity (higher than those of the other considered vegetable oils), with a good influence on emulsion stability (viscosity is a key parameter in emulsion stability). The authors suggested that the ionic liquid is adsorbed in the aqueous/organic interface along with span 80 (due to its amphipathic properties) and reduces as the internal phase droplets coalesce within the membrane phase. The membrane stability is increased by preventing the internal droplets merging, due to the strong interaction present between the ionic liquid and NaOH [115].

Matsumoto et al. (2007) used a supported liquid membrane consisting of an ionic liquid, 1-alkyl-3-methyl-imidazolium hexafluorophosphate, and a fixed porous support composed of polyvinylidene fluoride, with a thickness of 125 μm and a pore size of 0.45 μm, having an area of 12 cm^2^. Mixing of the two aqueous solutions was performed with the help of two magnetic stirrers with a speed of 300 rpm [116].

Martak et al. (2008) used a spiral module with a supported liquid membrane to separate lactic acid, using an ionic liquid (trihexyl-(tetradecyl) phosphonium bis 2,4,4-trimethylphosphinate, Cyphos IL-104), included in a polytetrafluoroethylene (PTFE) microporous film, 66.4 μm thick, with an average pore diameter of 0.2 μm and a porosity of approximately 70%, the supported membrane being arranged in a spiral mode [117]. The authors observed in this system the transport of water in the opposite direction to lactic acid by the formation of inverse micelles at the interface between the liquid membrane and the final aqueous solution. Based on the equilibrium data and this observation, a new separation mechanism has been proposed which includes 6 steps:

1. The destruction of inverse micelles at the interface of the initial aqueous phase and the liquid membrane
2. The formation of a hydrated complex between undissociated lactic acid, LAH and ionic liquid according to the following equation:
  $$p\;{\mathrm{LAH}\;}_{\left(\mathrm{aq}\right)}\;+2\;H_{2}O+{\mathrm{IL}}_{\left(\mathrm{org}\right)}\;↔\;{\left[{\left(\mathrm{LAH}\right)}_{p}\mathrm{IL}{\left(H_{2}O\right)}_{2}\right]}_{\left(\mathrm{org}\right)}$$
3. The transport of this complex through the liquid membrane
4. The decomposition of the complex at the interface between the membrane and the final aqueous phase according to this equation:
  $${\left[{\left(\mathrm{LAH}\right)}_{p}\mathrm{IL}{\left(H_{2}O\right)}_{2}\right]}_{\left(\mathrm{org}\right)}\;↔\;p\;{{\mathrm{LA}}^{-}}_{\left(\mathrm{ap}\right)}\;+\;3\;H_{2}O\;+\;{\mathrm{IL}}_{\left(\mathrm{org}\right)}$$
5. The formation of inverse micelles at the re-extraction interface between free molecules of ionic liquid, IL and water molecules
6. Their transport through the liquid membrane

The authors analyzed the mass transfer of lactic acid in this system. It was observed that although the distribution coefficient in the Cyphos IL-104/n-dodecane mixture is directly proportional to the carrier concentration, in the case of increasing Cyphos IL-104 concentration from 0.32 to 0.72 kmol/m^3^, no improvement in the global mass transfer coefficient was recorded. This was due to the increase in the membrane viscosity, and, implicitly, a slowed diffusion of the complex [(LAH)pIL(H_2_O)_2_]. Due to the positive effect of temperature on the decomposition rate of this compound, the variation from 25 °C to 35 °C caused the global mass transfer coefficient to increase from 50 to 70%. The stability and performance of the liquid membrane was maintained for 5.3 days [114].

Khan et al. (2021) developed an ionic liquid-based emulsion membrane (ILEMs) for lactic acid separation, analyzing three ionic liquids: tetramethylammonium chloride [TMAm] [Cl], tetramethylammonium acetate [TMAm] [Ac] and tributyl methylammonium chloride [TBMAm] [Cl], as a carrier dissolved in commercial grade olive oil and NaOH solution was used for re-extraction. For the stabilization of the liquid membrane, Tween 80 and Span 20 were used as emulsifiers, the membrane being stable for 134 min. The best results (94.50% lactic acid separation) were obtained for [TMAm] [Ac] in the following conditions: initial lactic acid concentration 0.05 M, 1.0 wt.% Span 20, 0.3 M NaOH, 0.3 wt.% [TMAm] [Ac], 250 rpm stirring speed, 25 min stirring and 15 min of settling time, 0.3 ratio of internal phase to diluent phase and 3:1 external phase to membrane phase ratio [118]. Kumar et al., 2018 used an environmentally friendly emulsion ionic liquid membrane using the ionic liquid: tri-n-octylmethylammonium chloride, [TOMAC], dissolved in a solvent mixture of rice bran oil and hexane in the volume ratio (70:30) for the removal of lactic acid from waste streams obtaining 90% extraction efficiency. The emulsion liquid membrane contained sodium hydroxide (NaOH) as stripping solution, 2.66 vol% Span-80 (emulsifying agent), 0.2 vol% [TOMAC] concentration, and was prepared at 2100 rpm emulsification speed and 20 min emulsification time and was stable for 90 min [119].

Baylan and Çehreli, 2018 analyzed a bulk ionic liquid membrane for levulinic acid (5–10% (*w*/*w*)) separation. The carrier used was tributyl phosphate (0–2 moll/L) dissolved in four different hydrophobic imidazolium-based ionic liquids: 1-Butyl-3-methylimidazolium bis(trifluoromethyl sulfonyl)imide [BMIM] [Tf2N], 1-Butyl-3-methylimidazolium hexafluoro phosphate [BMIM] [PF6], 1-Hexyl-3-methylimidazolium bis(trifluoromethyl sulfonyl)imide [HMIM] [Tf2N], 1-Hexyl-3-methylimidazolium hexafluorophosphate [HMIM] [PF6], and NaOH solutions (0–4 N) were used as stripping phase. The highest extraction efficiency (98.63% and 90.92 stripping efficiency) was obtained for [BMIM] [Tf2N] and 6.015%, *w*/*w* levulinic acid concentration, 2 mol/L TBP, 4 N NaOH concentration [120].

López-Porfiri et al., 2022 analyzed a green supported liquid membrane for succinic acid separation. The ionic liquid used was 1-butyl-1-methylpyrrolidinium bis(trifluoromethyl sulfonyl)imide introduced by impregnation into a porous support consisting of hydrophobic polyvinylidene fluoride (PVDF). The highest value: 1.510–6 m/s for membrane permeability was obtained for 0.5M NaOH (compared to 1.210–6 m/s for 0.1 M NaOH). The authors’ purpose for improving the SLM extraction capacity was a countercurrent configuration of 5-stages, with an estimated total mass transfer area of 719 m^2^ [121].

Baylan and Çehreli, 2019 used a bulk ionic liquid membrane for acetic acid separation. 1-Butyl-3-methylimidazolium bis (trifluoromethyl sulfonyl) imide [BMIM] [Tf2N], with low viscosity and high hydrophobicity, offered the best results (92% extraction efficiency and 80% stripping efficiency) for acetic acid removal in the following conditions: 2 mol/L TBP concentration and 4 N NaOH as stripping phase, 10% (*w*/*w*) acetic acid, and both interfaces. The feed/membrane phase surface area and membrane/stripping surface area were equal to 0.95 cm^2^ [122].

## 6. Conclusions and Future Perspectives

The efficient separation and concentration of a natural product, primary or secondary metabolite, is a complex problem that requires a detailed analysis of the structural features of the compound considered in order to choose an appropriate method. Due to the multitude and diversity, but also the special importance in food and human health, this category of compounds is continuously attracting the attention of scientists. In an attempt to develop new technologies for the replacement of fossil-based resources with renewable ones, the bio-synthetic production of organic acids (lactic, succinic, and butyric acid) has received increased attention. In order for these processes to be economically feasible, the separation step requires new successful and environmentally friendly approaches, as in industrial production, the downstream part occupies more than 50% of the production cost due carboxylic acid’s high affinity to water.

Extraction is an important separation technique in chemistry and biotechnology, being most often used as the first step in the recovery of a compound. It competes with many other separation methods, including: adsorption, precipitation, distillation, chromatography, crystallization, ion exchange, semipermeable membrane separation and electrodialysis. However, these processes can either be applied only intermittently or for temperature-resistant compounds or require a preliminary treatment of the mixture to be separated, which limits the fields of application and leads to an increase in price. In addition to these drawbacks, an important problem in the recovery of carboxylic acids is the complexity of the environment subjected to separation, especially in the case of fermentation broths containing a variety of by-products with physicochemical characteristics similar to those of the basic product, which are sometimes co-extracted. Considering the increasingly stringent requirements for the final product’s quality, the need to protect the environment and achieve high yields, separation processes are subject to increasing demands. As a solution, the use of highly selective and efficient ionic liquid as solvents in the extraction process has emerged.

Pertraction or extraction and transport through liquid membranes are one of the relatively new techniques applied for the separation of carboxylic acids. The use of extraction offers the possibility to overcome the limitations imposed by the direct contact between the organic and the aqueous phase specific to the extraction, namely the formation of stable emulsions and the need to regenerate the organic solvent while maintaining the advantages of continuous operation. The technological applications of this process for carboxylic acid recovery have developed rapidly, as a substantial part of the technological and financial success of biotechnological processes depends on the post-fermentation stages.

The solvent and carrier selection are, however, a challenge in both processes, as several characteristics need to be considered such as selectivity, solubility, cost and safety; hydrophobicity, density, polarity, viscosity, recoverability and environmental effects (the use of volatile organic solvents has a negative impact on the environment) are also important. Taking all these into consideration, ionic liquids are efficient alternatives to classical solvents. However, the selection of a suitable cation and anion combination from thousands of ILs possible combinations with excellent solvation properties is quite challenging. Analyzing the reviewed processes, in the case of reactive extraction, the use of green solvents—ionic liquids—is a sustainable alternative, as it provides high efficiency and does not affect the solute structure. However, the water co-extraction is a problem that needs to be solved in order to obtain concentrated extracts.

For pertraction, a considerable gap in all studies is related to implementing the technology in industrial size equipment. For SLM, this could be overcome, but for ELM and BLM, it is an important challenge. Additionally, for ELM, its poor stability is a main drawback for its large-scale industrial application.

Even if many researchers publish articles on reactive extraction and pertraction, highlighting several advantages, the technology is not yet close to industrial scale. Research should be focused on experiments using real fermentation broth; optimization studies are required for scale up.

Compared with conventional solvents, it can be concluded that ionic liquids are important alternatives to be considered for further improvement of the carboxylic acid separation process.

## Figures and Tables

**Table 1:** Industrial production and use of ionic liquids.

Companies Producing Ionic Liquids	Companies Using Ionic Liquids
Solvay	BASF (also a supplier of imidazolium-based ionic liquids)
Scionix	Eastman Chemical Company
Proionic	Sinopec
Iolitec	PetroChina
Solvionic	QUILL (Queen’s University Ionic Liquid Laboratories)

**Table 2:** Main ionic liquids physical properties used in biosynthetic compounds extraction [16,17,18,19].

Ionic Liquid	Density, g/mL, 25 °C	Viscosity, cP, 25 °C	Melting Point, °C
Trihexyl(tetradecyl)phosphonium decanoate, CYPHOS^®^ IL 103	0.89	319	24
Trihexyl(tetradecyl)phosphonium bis(2,4,4-trimethylpentyl)phosphinate, CYPHOS^®^ IL 104	0.895	805.8 (1198)	Not determined
Trihexyl(tetradecyl)phosphonium dicyanamide, CYPHOS^®^ IL 105	0.898	28.2–1646.8	Not determined
1-Butyl-3-methyl-imidazolium-hexafluorophosphate, [BMIM] [PF6]	1.367	274 (381)	6.5
1-Hexyl-3-methyl-imidazolium-hexafluorophosphate [HMIM] [PF6]	1.38	585	−73.5
1-Methyl-3-octyl-imidazolium-hexafluorophosphate [OMIM] [PF6]	1.24	682 (608)	−88

**Table 3:** Carboxylic acid extraction and reactive extraction with ionic liquids reported in the literature.

Carboxylic Acid	Ionic Liquid	Distribution Coefficient	Loading	Reference
Lactic acid	*Cyphos IL*-104, trihexyl-(tetradecyl)phosphonium bis 2,4,4-trimethylpentylphosphinate	40	2.4	[94]
	[P_4444_]Cl, tetrabutylphosphonium chloride, [P_444,14_]Cl, tributyltetradecylphosphonium chloride, [P_666,14_]Cl, trihexyltetradecylphosphonium chloride	6.08 2.71 3.28	2	[95]
	[P_66614_] [Cl], Tetradecyltrihexyl phosphonium chloride [P_66614_] [Dec], Tetradecyltrihexyl phosphonium decanoate [P_66614_] [Phos], Tetradecyltrihexyl phosphonium bis (2,4,4-trimethylpentyl) phosphinate	1.6 20.9 (two step extraction) 5	-	[96]
	1-butyl-3-methylimidazolium hexafluorophosphate	255	0.91	[97]
Butyric acid	*Cyphos IL*-104, trihexyl-(tetradecyl) phosphonium bis 2,4,4-trimethylpentylphosphinate	100 (45 °C)	3	[98]
	trialkylmethylammonium bis-(2,4,4-trimethylpentyl)phosphinate	5.47	7.12	[99]
Succinic acid	40 wt% [C_6_C_1_Im]Br—10 wt% (NH_4_)_2_SO_4_	1.06	85.5	[100]
	[P_6,6,6,14_] [PHOS] trihexyltetradecylphosphonium phosphinate	3.04 (78.4% extraction efficiency)		[101]
Glycolic Acid	1-butyl-3-methylimidazolium hexafluorophosphate	410	0.895	[102]
Valeric acid	1-hexyl-3-methylimidazolium hexafluorophosphate	7.31	0.26	[103]
Thioglycolic acid	[OMIM]OTf, 1-octyl-3- methyl-imidazolium trifluoromethanesulfonate	24.09	-	[104]
Levulinic acid	1-ethylpyridinium bis (trifluoromethylsulfonyl)imide, [Epy] [NTf_2_]	3.5	-	[105]
	BMIMPF_6_	1.05	-	[106]
Propionic acid	[HMIM] [PF_6_] [HMIM] [Tf_2_N]	Extraction efficiency 87.56% 88.16%		[71]
Protocatechuic acid	BMIM[TF_2_N] BMIM[PF_6_] MPPyr[Tf_2_N] MOA[Tf_2_N] CYPHOS IL-101 CYPHOS IL-104	0.16 0 0.11 0.12 47.1 54.2	-	[107]
Adipic acid	BMIM[TF_2_N] BMIM[PF_6_] MPPyr[Tf_2_N] MOA[Tf2N] CYPHOS IL-101 CYPHOS IL-104	0.06 0.05 0.0 0 14.7 2.25	-	[107]
Para-aminobenzoic acid	BMIM[TF_2_N] BMIM[PF_6_] MPPyr[Tf_2_N] MOA[Tf_2_N] CYPHOS IL-101 CYPHOS IL-104	0.94 4.39 0.61 0 22.7 3.7	-	[107]
Nicotinic acid	[C_6_mim]ClO_4_ 1-Hexyl-3-methylimidazolium perchlorate	22	-	[108]

## References

[1] Zhao X., Cai P., Sun C., Pan Y. (2019). Application of ionic liquids in separation and analysis of carbohydrates: State of the art and future trends. Trends Anal. Chem..

[2] Callegari D., Colombi S., Nitti A., Simari C., Nicotera I., Ferrara C., Mustarelli P., Pasini D., Quartarone E. (2021). Autonomous Self-Healing Strategy for Stable Sodium-Ion Battery: A Case Study of Black Phosphorus Anodes. ACS Appl. Mater. Interfaces.

[3] Asakawa M., Brown C.l., Pasini D., Stoddart J.F., Wyatt P.G. (1996). Enantioselective Recognition of Amino Acids by Axially-Chiral π-Electron-Deficient Receptors. J. Org. Chem..

[4] Baker G.A., Baker S.N., Pandey S., Bright F.V. (2005). An analytical view of ionic liquids. Analyst.

[5] Parajó J.J., Macário I.P.E., De Gaetano Y., Dupont L., Salgado J., Pereira J.L., Gonçalves F.J.M., Mohamadou A., Ventura S.P.M. (2019). Glycine-betaine-derived ionic liquids: Synthesis, characterization and ecotoxicological evaluation. Ecotoxicol. Environ. Saf..

[6] Gavhane R.J., Madkar K.R., Kurhe D.N., Dagade D.H. (2019). Room Temperature Ionic Liquids from Purine and Pyrimidine Nucleobases. ChemistrySelect.

[7] Jayachandra R., Reddy S.R. (2016). A remarkable chiral recognition of racemic Mosher’s acid salt by naturally derived chiral ionic liquids using ^19^F NMR spectroscopy. RSC Adv..

[8] Kirchhecker S., Esposito D. (2016). Amino acid based ionic liquids: A green and sustainable perspective. Curr. Opin. Green Sustain. Chem..

[9] Bodo E. (2021). Modelling biocompatible ionic liquids based on organic acids and amino acids: Challenges for computational models and future perspectives. Org. Biomol. Chem..

[10] Pandey D.K., Materny A., Kiefer J., Singh D.K. (2022). Quantification of the interactions in halide-anion-based imidazolium ionic liquids. J. Ion. Liq..

[11] Lei Z., Chen B., Koo Y.-M., MacFarlane D.R. (2017). Introduction: Ionic Liquids. Chem. Rev..

[12] Tullo A.H. (2020). The time is now for ionic liquids. C&En.

[13] Greer A.J., Jacquemin J., Hardacre C. (2020). Industrial Applications of Ionic Liquids. Molecules.

[14] de Jesus S.S., Filho R.M. (2022). Are ionic liquids eco-friendly?. Renew. Sustain. Energy Rev..

[15] Eno E.A., Louis H., Unimuke T.O., Gber T.E., Mbonu I.J., Ndubisi C.J., Adalikwu S.A. (2022). Reactivity, stability, and thermodynamics of para-methylpyridinium-based ionic liquids: Insight from DFT, NCI, and QTAIM. J. Ion. Liq..

[16] Jacquemin J., Husson P., Padua A.A.H., Majer V. (2006). Density and viscosity of several pure and water saturated ionic liquids. Green Chem..

[17] Fraser K.J., MacFarlane D.R. (2009). Phosphonium-Based Ionic Liquids: An Overview. Aust. J. Chem..

[18] Matsumoto M., Panigrahi A., Murakami Y., Kondo K. (2011). Effect of Ammonium- and Phosphonium-Based Ionic Liquids on the Separation of Lactic Acid by Supported Ionic Liquid Membranes (SILMs). Membranes.

[19] Zheng D., Hua D., Hong Y., Ibrahim A.-R., Yao A., Pan J., Zhan G. (2020). Functions of Ionic Liquids in Preparing Membranes for Liquid Separations: A Review. Membranes.

[20] Sowmiah S., Srinivasadesikan V., Tseng M.-C., Chu Y.-H. (2009). On the Chemical Stabilities of Ionic Liquids. Molecules.

[21] Han D., Row K.H. (2010). Recent Applications of Ionic Liquids in Separation Technology. Molecules.

[22] Zhou H., Chen L., Wei Z., Lu Y., Peng C., Zhang B., Zhao X., Wu L., Wang Y. (2019). Effect of Ionic Composition on Physicochemical Properties of Mono-Ether Functional Ionic Liquids. Molecules.

[23] Shukla S.K., Mikkola J.-P. (2020). Use of Ionic Liquids in Protein and DNA Chemistry. Front. Chem..

[24] Silva W., Zanatta M., Ferreira A.S., Corvo M.C., Cabrita E.J. (2020). Revisiting Ionic Liquid Structure-Property Relationship: A Critical Analysis. Int. J. Mol. Sci..

[25] Wang Y.-L., Li B., Sarman S., Mocci F., Lu Z.-Y., Yuan J., Laaksonen A., Fayer M.D. (2020). Microstructural and dynamical heterogeneities in ionic liquids. Chem. Rev..

[26] Halder P., Kundu S., Patel S., Ramezani M., Parthasarathy R., Shah K. (2019). A Comparison of Ionic Liquids and Organic Solvents on the Separation of Cellulose-Rich Material from River Red Gum. BioEnergy Res..

[27] Singh S.K., Savoy A.W. (2020). Ionic liquids synthesis and applications: An overview. J. Mol. Liq..

[28] Imperato G., Konig B., Chiappe C. (2007). Ionic green solvents from renewable resources. Eur. J. Chem..

[29] Hulsbosch J., de Vos D.E., Binnemans K., Ameloot R. (2016). Biobased ionic liquids: Solvents for a green processing industry?. ACS Sustain. Chem. Eng..

[30] Bao W., Wang Z., Li Y. (2003). Synthesis of chiral ionic liquids from natural amino acids. J. Org. Chem..

[31] Clavier H., Boulanger L., Audic N., Toupet L., Mauduit M., Guillemin J.C. (2004). Design and synthesis of imidazolinium salts derived from (L)-valine. Investigation of their potential in chiral molecular recognition. Chem. Commun..

[32] Parmentier D., Metz S.J., Kroon M.C. (2013). Tetraalkylammonium oleate and linoleate based ionic liquids: Promising extractants for metal salts. Green Chem..

[33] Kirchhecker S., Antonietti M., Esposito D. (2014). Hydrothermal decarboxylation of amino acid derived imidazolium zwitterions: A sustainable approach towards ionic liquids. Green Chem..

[34] Yue S., Wang P., Hao X., Zang S. (2017). Dual amino-functionalized ionic liquids as effic ient catalysts for carbonate synthesis from carbon dioxide and epoxide under solvent and cocatalyst-free conditions. J. CO_2_ Util..

[35] Huang Q., Jing G., Zhou X., Lv B., Zhou Z. (2018). A novel biphasic solvent of amino-functionalized ionic liquid for CO_2_ capture: High efficiency and regenerability. J. CO_2_ Util..

[36] Sintra T.E., Gantman M.G., Ventura S.P.M., Coutinho J.A.P., Wasserscheid P., Schulz P.S. (2019). Synthesis and characterization of chiral ionic liquids based on quinine, L-proline and L-valine for enantiomeric recognition. J. Mol. Liq..

[37] Handy S.T., Okello M., Dickenson G. (2003). Solvents from biorenewable sources: Ionic liquids based on fructose. Org. Lett..

[38] Poletti L., Chiappe C., Lay L., Pieraccini D., Polito L., Russo G. (2007). Glucose-derived ionic liquids: Exploring low-cost sources for novel chiral solvents. Green Chem..

[39] Kumar V., Pei C., Olsen C.E., Schaffer S.J.C., Parmar V.S., Malhotra S.V. (2008). Novel carbohydrate-based chiral ammonium ionic liquids derived from isomannide. Tetrahedron Asymmetry.

[40] Plaza P.G.J., Bhongade B.A., Singh G. (2008). Synthesis of chiral carbohydrate ionic liquids. Synlett.

[41] Jha A.K., Jain N. (2013). Synthesis of glucose-tagged triazolium ionic liquids and their application as solvent and ligand for copper (I) catalyzed amination. Tetrahedron Lett..

[42] Socha A.M., Parthasarathi R., Shi J., Pattathil S., Whyte D., Bergeron M., George A., Tran K., Stavila V., Venkatachalam S. (2014). Efficient biomass pretreatment using ionic liquids derived from lignin and hemicellulose. Proc. Natl. Acad. Sci. USA.

[43] Brzeczek-Szafran A., Erfurt K., Blacha-Grzechnik A., Krzywiecki M., Boncel S., Chrobok A. (2019). Carbohydrate ionic liquids and salts as all-in-one precursors for N-doped carbon. ACS Sustain. Chem. Eng..

[44] Reiß M., Brietzke A., Eickner T., Stein F., Villinger A., Vogel C., Kragl U., Jopp S. (2020). Synthesis of novel carbohydrate based pyridinium ionic liquids and cytotoxicity of ionic liquids for mammalian cells. RSC Adv..

[45] Sernaglia M., Blanco D., Hernández Battez A., Viesca J.L., González R., Bartolomé M. (2020). Two fatty acid anion-based ionic liquids—Part I: Physicochemical properties and tribological behavior as neat lubricants. J. Mol. Liq..

[46] Nordin N., Ismail M.H., Ramlee M.Z., Jalil M.A., Yong F.-S.J., Wang Y., Sidek N., Misran M., Suhana N., Manan A. (2022). An efficient and chemical oxidants-free protocol of synthesizing carboxylic acids from aldehydes catalyzed by the betaine-fatty acids ionic liquid derived from vegetable oil. Catal. Today.

[47] Jia H., Wang S., Wang Z., Wang Q., Jia H., Song L., Qin X., Fan F., Li Z., Huang P. (2022). Investigation of anionic group effects on the shale inhibition performance of fatty acid-based ionic liquids and their inhibition mechanism. Colloids Surf. A Physicochem. Eng. Asp..

[48] Arif R., Mir A.W., Shaheen A. (2022). Synthesis, aggregation behavior and drug-binding interactions of fatty acid-imidazolium-based surface-active ionic liquids. Chem. Phys. Lipids.

[49] Jia H., Wang S., Xu Y., Wang T., Zhang L., Song J., Zhang X., Song L., Jia H., Yan H. (2022). Systematic investigation on the abnormal surface and interfacial activity of fatty acid ionic liquids. Colloids Surf. A Physicochem. Eng. Asp..

[50] Gruzdev M.S., Shmukler L.E., Kudryakova N.O., Kolker A.M., Safonova L.P. (2018). Synthesis and properties of triethanolamine-based salts with mineral and organic acids as protic ionic liquids. J. Mol. Liq..

[51] Janković B., Manić N., Buchner R., Płowaś-Korus I., Pereiro A.B., Amado-González E. (2019). Dielectric properties and kinetic analysis of nonisothermal decomposition of ionic liquids derived from organic acid. Thermochim. Acta.

[52] Dong H., Pan J., Huang S., Sun P., Gao W. (2022). Target polishing of KDP crystals by organic acid-ionic liquid-in-oil microemulsions. JCIS Open.

[53] Piedade P.J., Kochańska E., Lukasik R.M. (2022). Biodegradable ionic liquids in service of biomass upgrade. Curr. Opin. Green Sustain. Chem..

[54] Cho C.W., Pham T.P.T., Zhao Y., Stolte S., Yun Y.S. (2021). Review of the toxic effects of ionic liquids. Sci. Total Environ..

[55] Skarpalezos D., Tzani A., Avraam E., Politidis C., Kyritsis A., Detsi A. (2021). Synthesis, structure-properties relationship and biodegradability assessment of novel protic ionic liquids. J. Mol. Liq..

[56] Verma S., Verma A., Mondal M., Prasad N.E., Srivastava J., Singh S., Verma J.P., Saha S. (2022). Drastic influence of amide functionality and alkyl chain length dependent physical, thermal and structural properties of new pyridinium-amide cation based biodegradable room temperature ionic liquids. J. Mol. Struct..

[57] Oulego P., Faes J., González R., Viesca J.L., Blanco D., Battez A.H. (2019). Relationships between the physical properties and biodegradability and bacteria toxicity of fatty acid-based ionic liquids. J. Mol. Liq..

[58] Mena I.F., Diaz E., Palomar J., Rodriguez J.J., Mohedano A.F. (2020). Cation and anion effect on the biodegradability and toxicity of imidazolium- and choline-based ionic liquids. Chemosphere.

[59] Neumann J., Steudte S., Cho C.-W., Thöming J., Stolte S. (2014). Biodegradability of 27 pyrrolidinium, morpholinium, piperidinium, imidazolium and pyridinium ionic liquid cations under aerobic conditions. Green Chem..

[60] Patra A., Abdullah S., Pradhan R.C. (2022). Review on the extraction of bioactive compounds and characterization of fruit industry by-products. Bioresour. Bioprocess..

[61] Mora-Pale M., Meli L., Doherty T.V., Linhardt R.J., Dordick J.S. (2011). Room Temperature Ionic Liquids as Emerging Solvents for the Pretreatment of Lignocellulosic Biomass. Biotechnol. Bioeng..

[62] Lee S.B. (2005). Analysis of solvation in ionic liquids using a new linear solvation energy relationship. J. Chem. Technol. Biotechnol..

[63] Yalcin D., Drummond C.J., Greaves T.L. (2020). Solvation properties of protic ionic liquids and molecular solvents. Phys. Chem. Chem. Phys..

[64] Wang X., Zhang S., Yao J., Li H. (2019). The Polarity of Ionic Liquids: Relationship between Relative Permittivity and Spectroscopic Parameters of Probe. Ind. Eng. Chem. Res..

[65] Nasirpour N., Mohammadpourfard M., Heris S.Z. (2020). Ionic liquids: Promising compounds for sustainable chemical processes and applications. Chem. Eng. Res. Des..

[66] Mosallanejad M.R., Khosravi-Nikou M.R., Shariati A. (2019). Separation of ethanol from n-decane-ethanol mixtures using imidazolium based ionic liquids. J. Chem. Thermodyn..

[67] Shen Y., Xu Y., Meng D., Chen Z., Li H., Zhang Y., Zhu Z., Gao J., Wang Y. (2021). Molecular mechanism and extraction performance evaluation of ionic liquids for extraction process of n-heptane/n-propanol. Sep. Purif. Technol..

[68] Esfahani H.S., Khoshsima A., Pazuki G. (2020). Choline chloride-based deep eutectic solvents as green extractant for the efficient extraction of 1-butanol or 2-butanol from azeotropic n-heptane + butanol mixtures. J. Mol. Liq..

[69] Zhang Z., Liu X., Yao D., Ma Z., Zhao J., Zhang W., Cui P., Ma Y., Zhu Z., Wang Y. (2021). Molecular kinetic extraction mechanism analysis of 1-butanol from n-heptane-1-butanol by choline-based DESs as extractants. J. Mol. Liq..

[70] Zhang Y., Zhang Q., Xin H., Lv M., Zhang Z. (2021). COSMO-RS prediction, liquid-liquid equilibrium experiment and quantum chemistry calculation for the separation of n-butanol and n-heptane system using ionic liquids. J. Chem. Thermodyn..

[71] Marták J., Athankar K., Liptaj T., Polakovič M., Schlosser S. (2021). Extraction equilibria of propionic Acid in systems with phosphonium phosphinate ionic liquid, dodecane, and water. J. Chem. Eng. Data.

[72] Mukherjee S., Negi D., Swarna M., Munshi N.B. (2021). Reactive extraction of butyric acid from water using trioctyl amine in 1-decanol and green natural oils. J. Chem. Eng. Data.

[73] Marták J., Liptaj T., Polakovič M., Schlosser S. (2021). New phosphonium ionic liquid with neodecanoate anion as butyric acid extractant. Chem. Pap..

[74] Lalikoglu M. (2022). Separation of butyric acid from aqueous media using menthol-based hydrophobic deep eutectic solvent and modeling by response surface methodology. Biomass Convers. Biorefinery.

[75] Tharani D., Ananthasubramanian M. (2021). Process intensification in separation and recovery of biogenic volatile fatty acid obtained through acidogenic fermentation of organics-rich substrates. Chem. Eng. Process.-Process Intensif..

[76] Zeng Q., Wang Y., Li N., Huang X., Ding X., Lin X., Huang S., Liu X. (2013). Extraction of proteins with ionic liquid aqueous two-phase system based on guanidine ionic liquid. Talanta.

[77] Li Y., Fang F., Sun M., Zhao Q., Hu Y., Sui Z., Liang Z., Zhang L., Zhang Y. (2020). Ionic liquid-assisted protein extraction method for plant phosphoproteome analysis. Talanta.

[78] Tarannum A., Rao J.R., Fathima N. (2022). Insights into protein-ionic liquid interaction: A comprehensive overview on theoretical and experimental approaches. Int. J. Biol. Macromol..

[79] Moattar M.T.Z., Shekaari H., Jafaro P. (2020). Structural effects of choline amino acid ionic liquids on the extraction of bovine serum albumin by green and biocompatible aqueous biphasic systems composed of polypropylene Glycol400 and choline amino acid ionic liquids. J. Mol. Liq..

[80] Phakoukaki Y.V., O’Shaughnessy P., Angeli P. (2022). Intensified liquid-liquid extraction of biomolecules using ionic liquids in small channels. Sep. Purif. Technol..

[81] Hasanov I., Shanmugam S., Kikas T. (2022). Extraction and isolation of lignin from ash tree (*Fraxinus excelsior*) with protic ionic liquids (PILs). Chemosphere.

[82] Kaur G., Kumar H., Singla M. (2022). Diverse applications of ionic liquids: A comprehensive review. J. Mol. Liq..

[83] Lidén G. (2017). Carboxylic Acid Production. Fermentation.

[84] Gonzalez-Garcia R.A., McCubbin T., Navone L., Stowers C., Nielsen L.K., Marcellin E. (2017). Microbial Propionic Acid Production. Fermentation.

[85] Llamas M., Greses S., Tomás-Pejó E., González-Fernández C. (2022). Carboxylic acids production via anaerobic fermentation: Microbial communities’ responses to stepwise and direct hydraulic retention time decrease. Bioresour. Technol..

[86] Karp E.M., Cywar R.M., Manker L.P., Saboe P.O., Nimlos C.T., Salvachúa D., Wang X., Black B.A., Reed M.L., Michener W.E. (2018). Post-fermentation recovery of biobased carboxylic acids. ACS Sustain. Chem. Eng..

[87] López-Garzón C.S., Straathof A.J.J. (2014). Recovery of carboxylic acids produced by fermentation. Biotechnol. Adv..

[88] Marták J., Schlosser S. (2019). Influence of anion and cation. Structure of ionic liquids on carboxylic acids extraction. Front. Chem..

[89] Shimizu K., Alves de Freitas A., Burba C.M. (2022). Cation-anion and cation-cation interactions in mixtures of hydroxy-functionalized and aprotic ionic liquids. J. Ion. Liq..

[90] Sprakel L.M.J., Schuur B. (2019). Solvent developments for liquid-liquid extraction of carboxylic acids in perspective. Sep. Purif. Technol..

[91] Weiß N., Schmidt C.H., Thielemann G., Heid E., Schröder C., Spange S. (2021). The physical significance of the Kamlet–Taft π* parameter of ionic liquids. Phys. Chem. Chem. Phys..

[92] Schlosser Š., Marták J., Blahušiak M. (2018). Specific phenomena in carboxylic acids extraction by selected types of hydrophobic ionic liquids. Chem. Papers.

[93] Sheridan Q.R., Schneider W.F., Maginn E.J. (2016). Anion dependent dynamics and water solubility explained by hydrogen bonding interactions in mixtures of water and aprotic heterocyclic anion ionic liquids. J. Phys. Chem. B.

[94] Marták J., Schlosser S. (2007). Extraction of lactic acid by phosphonium ionic liquids. Sep. Purif. Technol..

[95] Tonova K., Svinyarov I., Bogdanov M.G. (2015). Biocompatible ionic liquids in liquid–liquid extraction of lactic acid: A comparative study. Int. J. Chem. Nuclear Mater. Metall. Eng..

[96] Oliveira F.S., Araújo J.M.M., Ferreira R., Rebelo L.P.N., Marrucho I.M. (2012). Extraction of l-lactic, l-malic, and succinic acids using phosphonium-based ionic liquids. Sep. Purif. Technol..

[97] Çevik A., Aşçı Y.S., Lalikoglu M. (2022). Investigation of the effects of ionic liquid as diluent in separation of lactic acid from aqueous media by reactive extraction. Biomass Convers. Biorefinery.

[98] Marták J., Schlosser S. (2016). New Mechanism and model of butyric acid extraction by phosphonium ionic liquid. J. Chem. Eng. Data.

[99] Blahušiak M., Schlosser S., Marták J. (2013). Extraction of butyric acid with a solvent containing ammonium ionic liquid. Sep. Purif. Technol..

[100] Pratiwi A.I., Yokouchi T., Matsumoto M., Kondo K. (2015). Extraction of succinic acid by aqueous two-phase system using alcohols/salts and ionic liquids/salts. Sep. Purif. Technol..

[101] Zurob E., Rivas D., Olea F., Plaza A., Merlet G., Araya-López C., Romero J., Quijada-Maldonado E., Cabezas R. (2022). Succinic acid recovery from a glycerol-based solution using phosphonium ionic liquids supported by COSMO-RS. Fluid Phase Equilib..

[102] Aşçı Y.S. (2017). Examination of the efficiency of ionic liquids in glycolic acid separation from aqueous solution by using reactive extraction method. J. Turk. Chem. Soc. Sect. A Chem..

[103] Baylan N. (2019). Separation of valeric acid from aqueous solutions by reactive extraction using 1-hexyl-3-methylimidazolium hexafluorophosphate. Desalin. Water Treat..

[104] Zhou Y., Xu D., Zhang L., Ma Y., Ma X., Gao J., Wang Y. (2018). Separation of thioglycolic acid from its aqueous solution by ionic liquids: Ionic liquids selection by the COSMO-SAC model and liquid-liquid phase equilibrium. J. Chem. Thermodyn..

[105] Villar L., González B., Díaz I., Domínguez Á., González E.J. (2020). Role of the cation on the liquid extraction of levulinic acid from water using NTf2-based ionic liquids: Experimental data and computational analysis. J. Mol. Liq..

[106] Gök A. (2019). Experimental design of reactive extraction of levulinic acid using green solvents. J. Appl. Nat. Sci..

[107] De Brabander P., Uitterhaegen E., Verhoeven E., Vander Cruyssen C., De Winter K., Soetaert W. (2021). In situ product recovery of bio-based industrial platform chemicals: A guideline to solvent selection. Fermentation.

[108] Fan Y., Cai D., Yang L., Chen X., Zhang L. (2019). Extraction behavior of nicotinic acid and nicotinamide in ionic liquids. Chem. Eng. Res. Des..

[109] Tonova K. (2017). State-of-the-art recovery of fermentative organic acids by ionic liquids: An overview. Hung. J. Ind. Chem..

[110] Shah S.N., Ibrahim M., Mutalib A., Mohd Pilus R.B., Lethesh K.C. (2015). Extraction of naphthenic acid from highly acidic oil using hydroxide-based ionic liquids. Energy Fuels.

[111] Geng F., Zhang R., Wu L., Tang Z., Liu H., Liu H., Liu Z., Xu C., Meng X. (2022). High-efficiency separation and extraction of naphthenic acid from high acid oils using imidazolium carbonate ionic liquids. Chin. J. Chem. Eng..

[112] Imdad S., Dohare R.K. (2022). A critical review on heavy metals removal using ionic liquid membranes from the industrial wastewater. Chem. Eng. Process-Process Intensif..

[113] Zante G., Boltoeva M., Masmoudi A., Barillon R., Trébouet D. (2022). Supported ionic liquid and polymer inclusion membranes for metal separation. Sep. Purif. Rev..

[114] Zaulkiflee N.D., Ahmad A.L., Che Lah N.F. (2022). Removal of emerging contaminants by emulsion liquid membrane: Perspective and challenges. Environ. Sci. Pollut. Res..

[115] Avinash Thakur P., Jawa G.K. (2022). Comparative study on effect of ionic liquids on static stability of green emulsion liquid membrane. Colloids Surf. A Physicochem. Eng..

[116] Matsumoto M., Ohtani T., Kondo K. (2007). Comparison of solvent extraction and supported liquid membrane permeation using an ionic liquid for concentrating penicillin G. J. Membr. Sci..

[117] Martak J., Schlosser S., Vlckova S. (2008). Pertraction of lactic acid through supported liquid membranes containing phosphonium ionic liquid. J. Membr. Sci..

[118] Khan H.W., Reddy A.V.B.V., Bustam M.A., Goto M., Moniruzzaman M. (2021). Development and optimization of ionic liquid-based emulsion liquid membrane process for efficient recovery of lactic acid from aqueous streams. Biochem. Eng. J..

[119] Kumar A., Thakur A., Panesar P.S. (2018). Lactic acid extraction using environmentally benign green emulsion ionic liquid membrane. J. Clean. Prod..

[120] Baylan N., Çehreli S. (2018). Ionic liquids as bulk liquid membranes on levulinic acid removal: A design study. J. Mol. Liq..

[121] López-Porfiri P., González-Miquel M., Gorgojo P. (2022). Green supported liquid membranes: The permeability activity-based linear operation (PABLO) method. Chem. Eng. J..

[122] Baylan N., Çehreli S. (2019). Removal of acetic acid from aqueous solutions using bulk ionic liquid membranes: A transport and experimental design study. Sep. Purif. Technol..

